# Application of Inverse Finite Element Method to Shape Sensing of Curved Beams

**DOI:** 10.3390/s20247012

**Published:** 2020-12-08

**Authors:** Pierclaudio Savino, Francesco Tondolo, Marco Gherlone, Alexander Tessler

**Affiliations:** 1 Department of Structural, Geotechnical and Building Engineering, Politecnico di Torino, Corso Duca degli Abruzzi 24, 10129 Torino, Italy; pierclaudio.savino@polito.it (P.S.); francesco.tondolo@polito.it (F.T.); 2 Department of Mechanical and Aerospace Engineering, Politecnico di Torino, Corso Duca degli Abruzzi 24, 10129 Torino, Italy; marco.gherlone@polito.it; 3 Structural Mechanics and Concepts Branch, NASA Langley Research Center, Mail Stop 190, Hampton, VA 23681-2199, USA

**Keywords:** curved beam, iFEM, structural health monitoring, shape sensing

## Abstract

Curved beam, plate, and shell finite elements are commonly used in the finite element modeling of a wide range of civil and mechanical engineering structures. In civil engineering, curved elements are used to model tunnels, arch bridges, pipelines, and domes. Such structures provide a more efficient load transfer than their straight/flat counterparts due to the additional strength provided by their curved geometry. The load transfer is characterized by the bending, shear, and membrane actions. In this paper, a higher-order curved inverse beam element is developed for the inverse Finite Element Method (iFEM), which is aimed at reconstructing the deformed structural shapes based on real-time, in situ strain measurements. The proposed two-node inverse beam element is based on the quintic-degree polynomial shape functions that interpolate the kinematic variables. The element is C^2^ continuous and has rapid convergence characteristics. To assess the element predictive capabilities, several circular arch structures subjected to static loading are analyzed, under the assumption of linear elasticity and isotropic material behavior. Comparisons between direct FEM and iFEM results are presented. It is demonstrated that the present inverse beam finite element is both efficient and accurate, requiring only a few element subdivisions to reconstruct an accurate displacement field of shallow and deep curved beams.

## 1. Introduction

Civil engineering structures are commonly exposed to a series of loading and environmental conditions that impair their structural performance, integrity, and durability. The direct implications are detrimental social, environmental, and economic impacts. In this context, modern technologies that fall into the general category of Structural Health Monitoring (SHM) can potentially detect real-time information related to on-site structural conditions. The detection of unusual structural behavior not only contributes to reducing the uncertainty related to the monitored structure but also allows improving the efficiency of maintenance procedures. Therefore, it is necessary to develop an algorithm suitable for SHM that uses measured data by installed sensors. Such an algorithm should be robust, computationally stable, and accurate under a wide range of loads, material systems, and boundary conditions. This is of particular interest for civil engineering structures such as bridges and dams, as well as ships, aerospace vehicles, and many others. A key technology for SHM is commonly referred to as “shape sensing”, which allows real-time reconstruction of the structural displacements, strains, and stresses using a network of strain sensors. 

A novel algorithm that is well suited for SHM was originally developed by Tessler and Spangler [1,2,3]. The methodology, called the inverse Finite Element Method (iFEM), is formulated on the basis of a least-squares variational principle. The iFEM approach is general enough to model a wide variety of structures and is aimed at reconstructing the full-field displacements and strains based on in situ, discrete strain measurements. The formulation is based upon the minimization of a least squares functional that defines the error between analytic and measured section strains. Unlike other inverse methods, the iFEM algorithm involves only strain–displacement relations, and therefore, the structural response can be reconstructed without the knowledge of mechanical properties and loading conditions. Once the displacements and strains are reconstructed, the stresses can be computed using the relevant constitutive relations. The iFEM approach can provide accurate, stable, and fast solutions for any type of structural domain discretized by beam, frame, plate, or shell inverse finite elements. Originally, Tessler and Spangler [2] employed Mindlin (first-order shear deformation) theory as the kinematic basis for iFEM, and they developed a three-node triangular inverse shell element (iMIN3) to model plate and shell structures. Cerracchio et al. [4] applied the iMIN3 element to model composite stiffened shell structures subject to mechanical and thermal loads. Kefal et al. [5] developed a simple and efficient four-node quadrilateral inverse-shell element (iQS4). The iFEM approach has been further extended to multilayered composite and sandwich plates by Cerracchio et al. [6] and Kefal et al. [7,8]. Gherlone et al. [9,10] studied the shape-sensing of truss, beam, and frame structures by way of a 3D inverse beam element based on Timoshenko beam theory. More recently, Savino et al. [11] developed an iFEM formulation for beam and frame structures that behave according to Bernoulli–Euler beam theory with a consequent reduction of input strain data. To assess the robustness and accuracy of iFEM, various experimental and numerical studies have been performed for aerospace structures by Tessler and Spangler [2], Tessler [12], Tessler et al. [13], Quach et al. [14], Gherlone et al. [15], and for marine structures by Kefal et al. [16], Kefal and Oterkus [17,18].

The main focus of the present work is to extend the library of iFEM inverse elements and to develop an inverse curved beam element. Curved beams are widely used in a variety of practical applications such as arches, arch bridges, highway construction, tunnels, circumferential stiffeners, airplane wings, blades, and springs. Such structures are commonly modeled using curved finite elements. Due to their initial curvature, curved beams are more efficient in transferring the loads than straight beams, since the transfer occurs through the combined action of bending and membrane stiffnesses. Importantly, coupling between the bending and axial deformations is a source of difficulty in solving the governing equations. Many approaches in the literature have addressed the modeling issues associated with the bending–membrane coupling in curved finite elements. In particular, considerable attention has been devoted to developing suitable element shape functions for curved finite elements that include proper representations of rigid-body modes, bending–membrane coupling, and membrane and shear locking stiffening effects. Consequently, many efforts have focused on the choice of shape functions that best represent the curved beam behavior. The simplest modeling strategy for curved beams is an assembly of relatively short straight beam elements (Cyrus et al. [19], Kikuchi [20]). Such an approach generally requires a large number of elements to obtain converged solutions. To obtain an accurate solution independent on the number of subdivisions, it is necessary to take into account the curvature effect by solving the governing differential equations. Ashwell and Sabir [21] discussed the use and limitations of shape functions with and without explicit terms of rigid-body displacements, with varying depth of the arches. Convergent solutions using a low number of elements were obtained using thick arches and shape functions with explicit terms of rigid-body motions. Ashwell et al. [22] proposed an element based on simple strain functions that deals satisfactorily with arches of all proportions. By integrating the strain–displacement equations, new shape functions were obtained containing terms expressing rigid-body displacements and deformation components. It is now widely recognized that to satisfy the convergence condition and to have good accuracy, an adequate representation not only of the strain-inducing motions but also of strain-free motions is necessary (Dawe [23]). Davis et al. [24] presented a constant curvature beam finite element starting not from the displacement assumptions but from an integration of the exact differential equations of an infinitesimal element in equilibrium. Dawe [25,26] investigated different curved beam finite elements, up to the quintic polynomial order, based on independently interpolated displacement components. Computational studies have demonstrated that using higher-order polynomials for both the radial and circumferential displacements guarantees improved predictions even without the need to explicitly represent the rigid-body displacements. Meck [27] showed that it is not sufficient that the displacement function satisfies only the boundary conditions, but it should also satisfy the coupling between normal and tangential displacements. He suggested the use of an independent interpolation of a higher order for the radial displacement than the circumferential one to take into account the coupling and obtain good results. However, the formulation based on displacement fields often leads to excessively stiff behavior in thin regimes. In such analyses, the phenomenon of shear and membrane locking takes place when lower order shape functions are used. Therefore, much attention has been focused to overcome the locking phenomena. Stolarki and Belytschko [28] showed a complex interdependence between shear and membrane under-integration. Hybrid-stress formulations aimed to avoiding locking were explored as an alternative method based on equilibrium equations, constitutive relations, and variation of the related energy function (Stolarki and Belytschko [28], Saleeb [29]). Saffari and Tabatabaei [30], to avoid shear and membrane locking phenomena, considered the curved-beam finite element formulation by the trigonometric function for curvature. Gimena et al. [31] proposed a new system of twelve differential equations expressed in the global Cartesian coordinate system to simulate the structural behaviour of a general curved beam element. The lower-triangular form permits the determination of analytical results through successive simple integrations row by row. More recently, Tufekci et al. [32] presented a finite element formulation for in-plane static problems of curved beams with a continuously varying curvature and cross-section using the exact solution of the governing differential equations of in-plane behavior of curved beams defined by Tufekci and Arpaci [33].

To achieve accurate curved beam elements capable of rigid-body motion and locking-free behavior, higher-order polynomials are used to interpolate the element kinematics. In this study, the iFEM theory is based upon the minimization of a least-squares functional using the kinematic assumptions of Bernoulli–Euler curved beam theory that accounts for the membrane and bending deformations. The curved beam element is initially formulated in a local curvilinear coordinate system and subsequently transformed into a global Cartesian coordinate system to enable the curved beam structures to be analyzed. The accuracy of the inverse element is assessed by way of several numerical examples: (a) shallow, thick arch, (b) shallow, thin arch, (c) deep, thick arch, and (d) deep, thin arch. To simulate the experimentally measured strains and to provide an accurate reference solution for displacements and strains, high-fidelity FEM solutions are obtained using Bernoulli–Euler beam elements. For the inverse FEM, the effects of mesh refinement are also studied.

## 2. Governing Equations of the Elastic Theory

**Figure 1:**
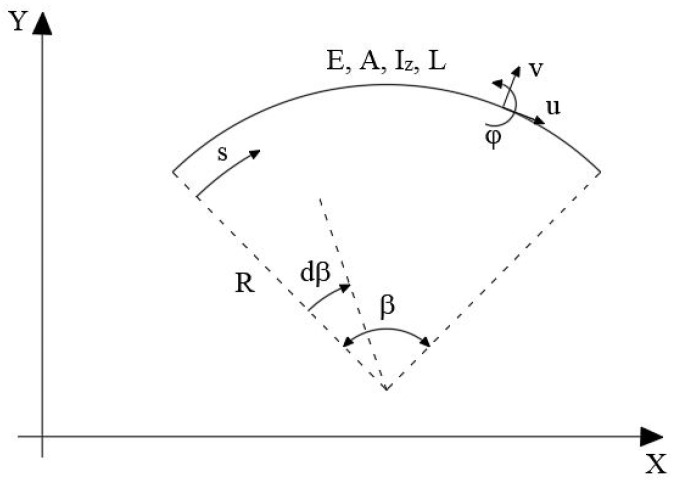
Curved beam geometry.

The geometry of a planar curved beam under consideration is depicted in Figure 1. The curved beam is defined by the Young’s modulus E, moment of inertia about the z axis I, cross-sectional area A, and length L.

The differential element of the axial beam coordinate ds can be readily defined using the curvilinear coordinates R and β (defined positive in the clockwise direction) by the equation

(1) ds=R·dβ

where R is the constant curvature radius.

The kinematics of every cross-section of the beam can be described by the circumferential displacement u (tangential to the s coordinate), radial displacement v (orthogonal to the s coordinate), and rotation φ positive in the counter clockwise direction (refer to Figure 1). Due to the intrinsic curvature, the kinematic equations take into account further angle variation, axial expansion, and curvature variation, which lead to the following generalized strain–displacement relations

(2) $$\gamma =-\frac{\mathrm{dv}}{\mathrm{ds}}+\frac{u}{R}+\varphi$$

(3) $$\varepsilon =\frac{\mathrm{du}}{\mathrm{ds}}+\frac{v}{R}$$

(4) $$\chi =\frac{d\varphi }{\mathrm{ds}}.$$

Assuming the Bernoulli–Euler hypothesis that neglects shear deformation (γ = 0), and introducing Equation (1), the two non-zero strain components become

(5) $$\varepsilon =\frac{1}{R}\left(\frac{\mathrm{du}}{d\beta }+v\right)$$

(6) $$\chi =\frac{1}{R^{2}}\left(-\frac{\mathrm{du}}{d\beta }+\frac{d^{2}v}{{d\beta }^{2}}\right).$$

It can be seen that in the curved beam analysis, the two displacements u and v are coupled within the strain–displacement relations.

The section strains that define the kinematic model are contained in the vector (henceforth, bold free letters will denote matrices and vectors)

(7) $$e\left(u\right)={\left\{\varepsilon ,\;\chi \right\}}^{T}.$$

The constitutive relations between the stress resultants and strain for a curvilinear beam with linear elastic behavior are

(8) N=EA·ε

(9) M=EI·χ

where EA is the axial stiffness and EI is the bending stiffness, N is the tangential force, and M is the bending moment.

Considering an infinitesimal length of a curved beam ds, subjected to both axial (p) and orthogonal (q) distributed loads, the tangential, radial, and rotational equilibrium equations are given by

(10) $$\frac{1}{R}\frac{\mathrm{dN}}{d\beta }+\frac{T}{R}+p=0$$

(11) $$\frac{1}{R}\frac{\mathrm{dT}}{d\beta }-\frac{N}{R}+q=0$$

(12) $$\frac{1}{R}\frac{\mathrm{dM}}{d\beta }-T=0$$

where the independent variable is the angular coordinate β, and T is the radial shear force. The membrane-bending interaction is clearly manifested in the equilibrium equations.

## 3. Inverse Finite Element Method for Curved Beams

In this section, the general framework of the iFEM approach is presented. The iFEM methodology reconstructs the deformed shape of a structure by minimizing a weighted least-squares functional **Φ** with respect to the unknown degrees of freedom (DOF). For a single element, the error functional between the section strains obtained by in situ strains measurements and analytical section strain is expressed by

(13) $$\Phi^{e}\left(u\right)={\left|\left|\varepsilon \left(u\right)-\varepsilon^{\varepsilon }\right|\right|}^{2}+{\left|\left|\chi \left(u\right)-\chi^{\varepsilon }\right|\right|}^{2}$$

where the squared error norms corresponding to the membrane and bending deformations are given as

(14) $${\left|\left|\varepsilon \left(u\right)-\varepsilon^{\varepsilon }\right|\right|}^{2}=\frac{L^{e}}{n}\cdot \overset{n}{\underset{i=1}{\sum }}{\left(\varepsilon \left(\beta_{i}\right)-\varepsilon_{i}^{\varepsilon }\right)}^{2}$$

(15) $${\left|\left|\chi \left(u\right)-\chi^{\varepsilon }\right|\right|}^{2}=\frac{I_{z}^{e}{\;L}^{e}}{A^{e}\;n}\cdot \overset{n}{\underset{i=1}{\sum }}{\left(\chi \left(\beta_{i}\right)-\chi_{i}^{\varepsilon }\right)}^{2}$$

where L^e^, A^e^, and $I_{z}^{e}$ are the length of the element, the cross-sectional area, and moment of inertia with respect to the z-axis of the section (see Figure 1), and n is the number of locations also named as “station points” where the section strains are evaluated with coordinates β_i_.

The kinematic variables **u** are interpolated within a finite element using a set of suitable shape functions **N**(β)

(16) $$u\left(\beta \right)=N\left(\beta \right)\cdot u^{e}$$

where **u**^e^ denotes the nodal DOF of the element consistent with respect to the order of interpolation. Thus, in the case of discretization with m elements, the total least squares functional is given by the sum

(17) $$\Phi =\overset{m}{\underset{e=1}{\sum }}\Phi^{e}$$

Substituting Equation (16) into Equations (14) and (15) gives the analytic section strains in terms of the nodal DOF

(18) $$e\left(u\right)=B\left(\beta \right)\cdot u^{e}$$

where the matrix **B**(β) contains the derivatives of the shape functions **N**(β). Considering in Equations (14) and (15) the section strain in analytical form, and minimizing the element functional (Equation (13)) with respect to the nodal DOF, leads to the inverse element matrix equation

(19) $$S^{e}\cdot u^{e}=h^{e}$$

where the matrix **S^e^** depends on the strain sensors locations, whereas the vector **h^e^** is a function of the measured section-strain values (Savino et al. [11]). Then, the local matrices of the discretized structure are assembled into a global linear system of equations, performing the usual finite element assembly operations and transformations into a global coordinate system, resulting in

(20) S·u=h.

By prescribing problem-specific displacement boundary conditions, a non-singular system matrix provides the solution for the unknown nodal DOF. Since for a given distribution of strain sensors, **S** remains unchanged, it needs to be inverted only once during the monitoring process. Consequently, Equation (20) can be solved for the unknown displacement DOF vector, u, very efficiently. However, the **h** vector is dependent on the measured strain values; thus, it needs to be updated during any deformation cycle. The key aspect of the inverse element formulation is the choice of suitable shape functions. In addition, a suitable number and location of strain sensors and their locations along the structure have to be established. Finally, the reconstructed displacement field along the individual inverse elements enables the direct calculations of element strains and stresses.

## 4. Element Shape Functions

**Figure 2:**
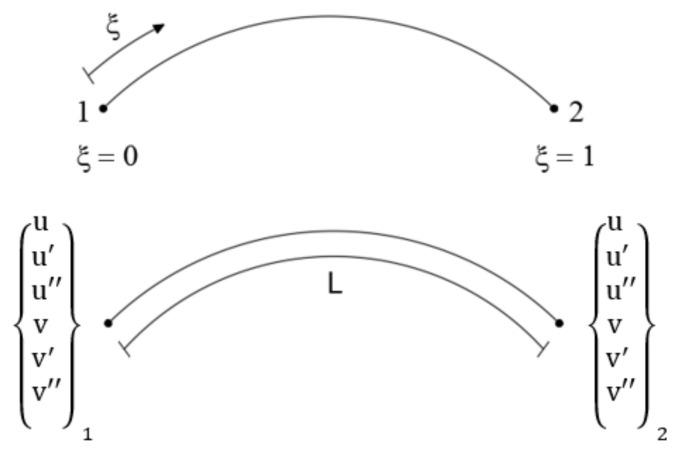
Two node inverse finite element.

The inverse curved element developed herein uses quintic polynomials to interpolate the displacement variables to avoid problems of rigid body motions or locking. The initial configuration of the element has at the two end nodes six DOF, which are u and v and their first and second-order derivatives (Figure 2).

The vector of nodal DOF is expressed as

(21) $$u^{e}={\left\{u_{1},u_{1}^{\prime },u_{1}^{\prime \prime },v_{1},v_{1}^{\prime },v_{1}^{\prime \prime },u_{2},u_{2}^{\prime },u_{2}^{\prime \prime },v_{2},v_{2}^{\prime },v_{2}^{\prime \prime }\right\}}^{T}.$$

The displacement interpolations are expressed as the second order Hermite polynomials given in terms of non-dimensional coordinates ξ ∈ [0, 1]

(22) $$u\left(\xi \right)=\overset{2}{\underset{i=1}{\sum }}(H_{0i}^{\left(2\right)}\left(\xi \right)\cdot u_{i}+H_{1i}^{\left(2\right)}\left(\xi \right)\cdot u_{i}^{\prime }+H_{2i}^{\left(2\right)}\left(\xi \right)\cdot u_{i}^{\prime \prime })$$

(23) $$v\left(\xi \right)=\overset{2}{\underset{i=1}{\sum }}(H_{0i}^{\left(2\right)}\left(\xi \right)\cdot v_{i}+H_{1i}^{\left(2\right)}\left(\xi \right)\cdot v_{i}^{\prime }+H_{2i}^{\left(2\right)}\left(\xi \right)\cdot v_{i}^{\prime \prime })$$

where $H_{\mathrm{ki}}^{\left(2\right)}$ (i = 1, 2; k = 0, 1, 2) are the quintic Hermite polynomials (Appendix A). Thus, continuity up to the second derivative is ensured between two adjacent elements. From Equations (5) and (6), it can be seen that these shape functions lead to a quintic membrane strain and a quartic curvature. Therefore, a minimum of five station points (i.e., strain sensor positions) for each inverse element are required to have consistency with respect to the bending curvature. In the present study, the minimum number of station points is used.

## 5. Section Strain Measurements

**Figure 3:**
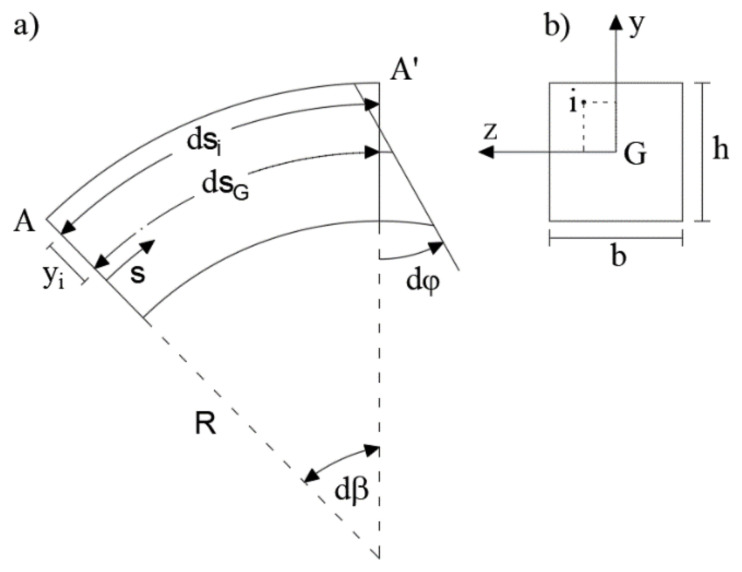
(**a**) Curved beam segment; (**b**) Cross-section.

A key step in the iFEM implementation is the evaluation of the experimental section strains from strain-sensor measurements. Taking into account a centroid reference system of the generic cross-section (Figure 3b), the fiber deformation in the point (z_i_, y_i_) needs to be defined (Figure 3a). Referring to the beam differential element between sections A and A’ (Figure 3a), the length of an undeformed fiber located at (z_i_, y_i_) is represented by

(24) $${\mathrm{ds}}_{i}=\left(R+y_{i}\right)d\beta$$

and the length of the centroidal fiber is given by

(25) $${\mathrm{ds}}_{G}=\mathrm{Rd}\beta .$$

Starting from the assumption that plane sections remain plane and using the principle of the superposition effect, the cross-sectional strains are given by both the axial and bending strain contributions.

Considering only a planar beam problem, i.e., cylindrical bending in the (y, s) plane, the fiber lengths in the deformed configuration is given by

(26) $${d\bar{s}}_{G}={\mathrm{ds}}_{G}+\varepsilon_{0}{\mathrm{ds}}_{G}$$

(27) $${d\bar{s}}_{i}={\mathrm{ds}}_{i}+{d\bar{s}}_{G}-{\mathrm{ds}}_{G}-y_{i}d\varphi$$

where the overmarked quantities depict the final lengths and ε_0_ is the centroidal axial strain.

Consequently, the fiber deformation at the point (z_i_, y_i_) is obtained by the equation

(28) $$\varepsilon_{s,i}=\frac{{d\bar{s}}_{i}-{\mathrm{ds}}_{i}\;}{{\mathrm{ds}}_{i}}=\frac{\varepsilon_{0}{\mathrm{ds}}_{G}-y_{i}d\varphi }{\left(R+y_{i}\right)d\beta }=\frac{R}{R+y_{i}}\left(\varepsilon_{0}-y_{i}\frac{d\varphi }{{\mathrm{ds}}_{G}}\right).$$

Assuming that the length of the beam segment equals the length of the centroidal fiber, the curvature can be defined according to Equation (4)

(29) $$\chi =\frac{d\varphi }{{\mathrm{ds}}_{G}}.$$

Substituting Equation (29) into Equation (18), the following equation for the generic fiber can be obtained

(30) $$\varepsilon_{s}=\frac{R}{R+y}\left(\varepsilon_{0}-y\chi \right).$$

Considering a configuration of two strain sensors placed on the external surface for each cross-section (±h/2), the following system of equations is readily solved to obtain the experimental section strains

(31) $$\left\{\begin{array}{l} \varepsilon_{s,\sup}^{*}=\frac{R}{R+\frac{h}{2}}\left(\varepsilon_{0}^{\varepsilon }-\frac{h}{2}\chi^{\varepsilon }\right)\\ \varepsilon_{s,\inf}^{*}=\frac{R}{R-\frac{h}{2}}\left(\varepsilon_{0}^{\varepsilon }+\frac{h}{2}\chi^{\varepsilon }\right) \end{array}\right.$$

where $\varepsilon_{s,\sup}^{*}$ and $\varepsilon_{s,\inf}^{*}$ represent the input strain from the i-th strain sensor at location y_i_, while $\varepsilon_{0}^{\varepsilon }$ and $\chi^{\varepsilon }$ are the experimental section strains for the plane beam problem.

## 6. Numerical Examples

The study is focused on the mesh convergence and the influence of the membrane-bending terms. The element formulation is first validated using a single curved member with a constant curvature for a combination of slenderness ratio (R/h) and subtended angle (β), according to the classification reported in Krishnan and Suresh [34]:

1. A thick, shallow arch clamped at one end
2. A thin, shallow arch clamped at one end
3. A thick, deep arch clamped at two ends
4. A thin, deep arch clamped at two ends.

The iFEM reconstruction analysis uses the input strain data obtained by the direct FEM analysis performed with LUSAS software [35]. Subsequently, the iFEM predictions are assessed by the average percent difference between the predicted and reference FEM (“computational experiment”) quantities, given as

(32) $$\mathrm{PD}\left(x\right)=100\%\left(\frac{1}{n}\overset{n}{\underset{i=1}{\sum }}\frac{x_{i}^{\mathrm{iFEM}}-x_{i}^{\mathrm{FEM}}}{x_{\max}^{\mathrm{FEM}}}\right)$$

where x indicates the quantity of interest and n indicates the number of the output points. The direct FEM model is composed of 20 beam elements based on Bernoulli–Euler theory. The element used is a parabolically curved beam element in which the tangential and radial displacements are approximated along the element as quadratic and cubic functions, respectively. The beam is modeled using a linear elastic isotropic material with Young’s modulus E = 30 GPa and Poisson’s ratio υ = 0.2.

### 6.1. A Thick, Shallow aRch Clamped at One End

**Figure 4:**
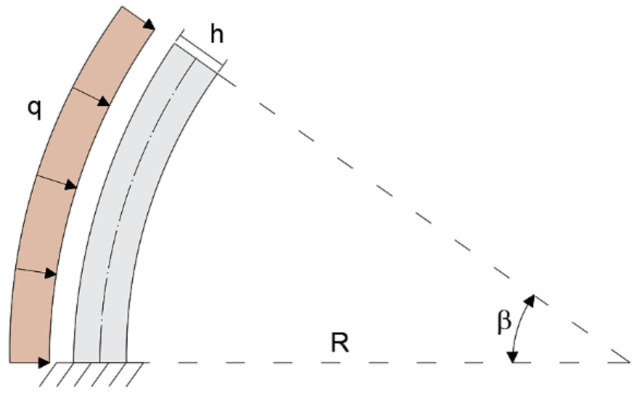
Boundary conditions of the shallow–thick arch.

The configuration shallow-thick arch is validated by considering a curved beam of R = 5m, opening angle of β = 35°, and rectangular cross-section with b = 0.3 m and h = 0.5 m (R/h = 10). The load q = 1 KN/m is applied uniformly along the entire length of the member (Figure 4).

**Figure 5:**
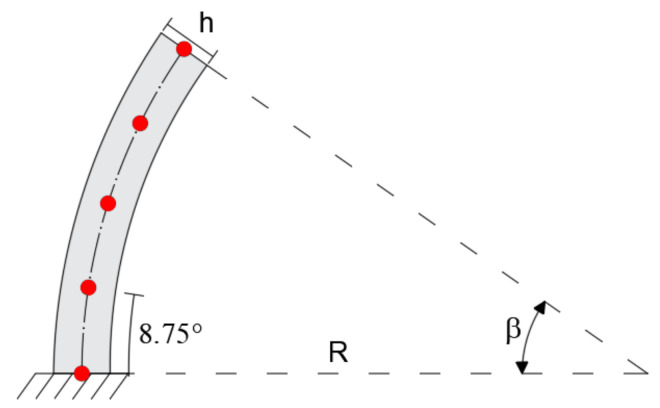
Inverse finite element.

In the iFEM analysis, the beam is modeled using a single inverse element restrained with the boundary conditions described above and five station points spaced by 8.75° (Figure 5).

**Figure 6:**
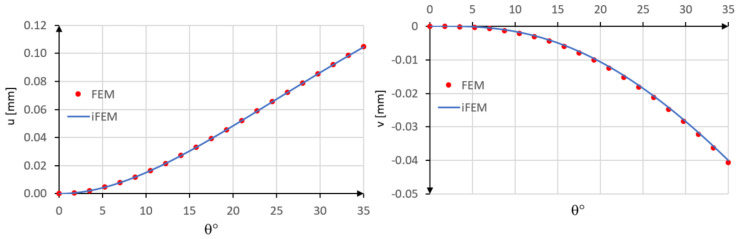
Comparison between inverse Finite Element Method (iFEM) and FEM results: u and v displacements.

The tangential u and radial v displacements are compared with those obtained using the direct FEM analysis. As it can be seen from the diagrams reported in Figure 6, a good agreement is achieved using only one inverse element.

The accuracy of the method is evaluated also in terms of percent differences for the two displacement components with PD(u) = 0.02% and PD(v) = 0.81%.

### 6.2. A Thin, Shallow Arch Clamped at One End

**Figure 7:**
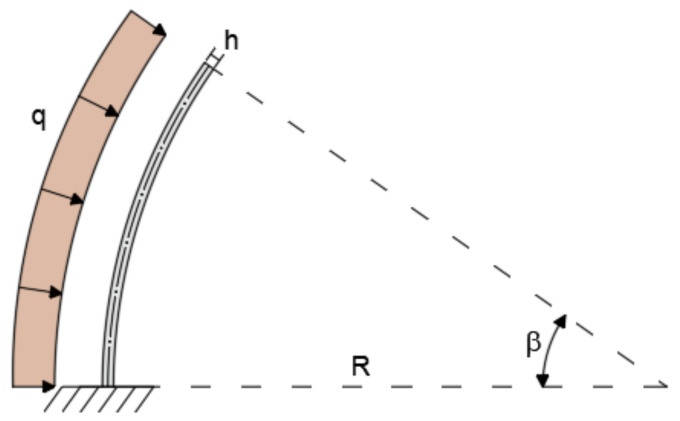
Boundary conditions of the shallow, thin arch.

The shallow moderately thin arch is studied using a curved beam with radius of curvature R = 5m, opening angle of β = 35°, and rectangular cross-section with the base b = 0.3 and height h = 0.1 m (R/h = 50). The remaining properties and boundary conditions shown in Figure 7 are the same as in the case of the shallow, thick arch example.

**Figure 8:**
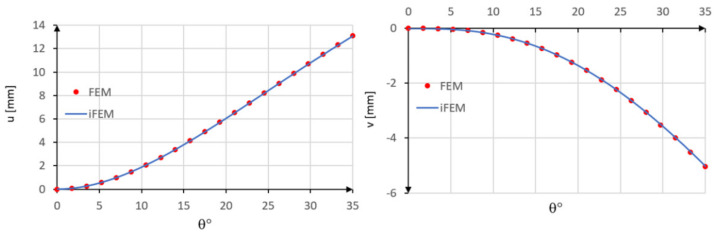
Comparison between iFEM and FEM results.

In this example, the iFEM analysis is performed using only a single inverse element with five station points spaced by 8.75° (Figure 5). The comparison between the iFEM displacements with those obtained by the direct FEM analysis shows the high accuracy of the present element (Figure 8).

The low difference percentage is confirmed also in this case for both tangential PD(u) = 0.06% and radial PD(v) = 0.09% displacements.

### 6.3. A Thick, Deep aRch Clamped at Both Ends

**Figure 9:**
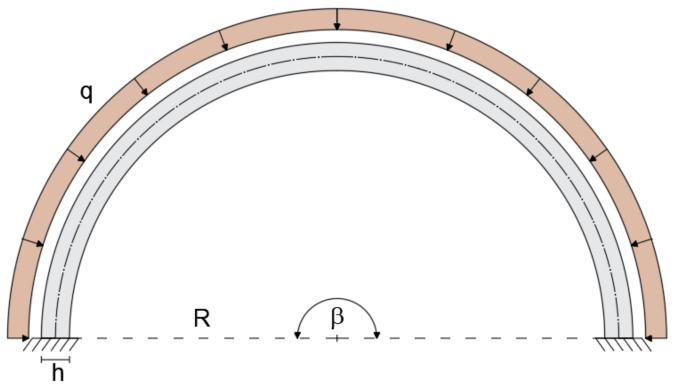
Geometry, loading, and boundary conditions of the thick, deep arch.

The deep curved arch is considered to assess the applicability of the present element to model an arch with a large subtended angle. The deep arch is modeled with a curved beam clamped at each end, subjected to uniformly distributed load q = 1 KN/m. The beam is considered to have a radius R = 5 m, an opening angle β = 180°, and a rectangular cross-section with height h = 0.5 m (R/h = 10) and width b = 0.3 m (Figure 9). The mechanical properties remain unchanged with respect to the previous cases.

**Figure 10:**
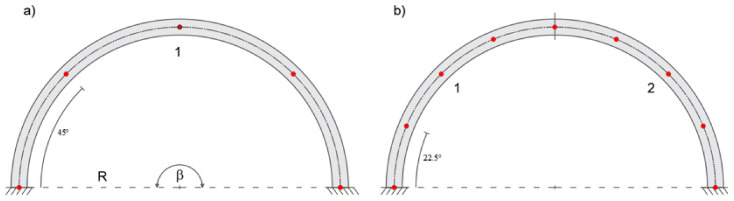

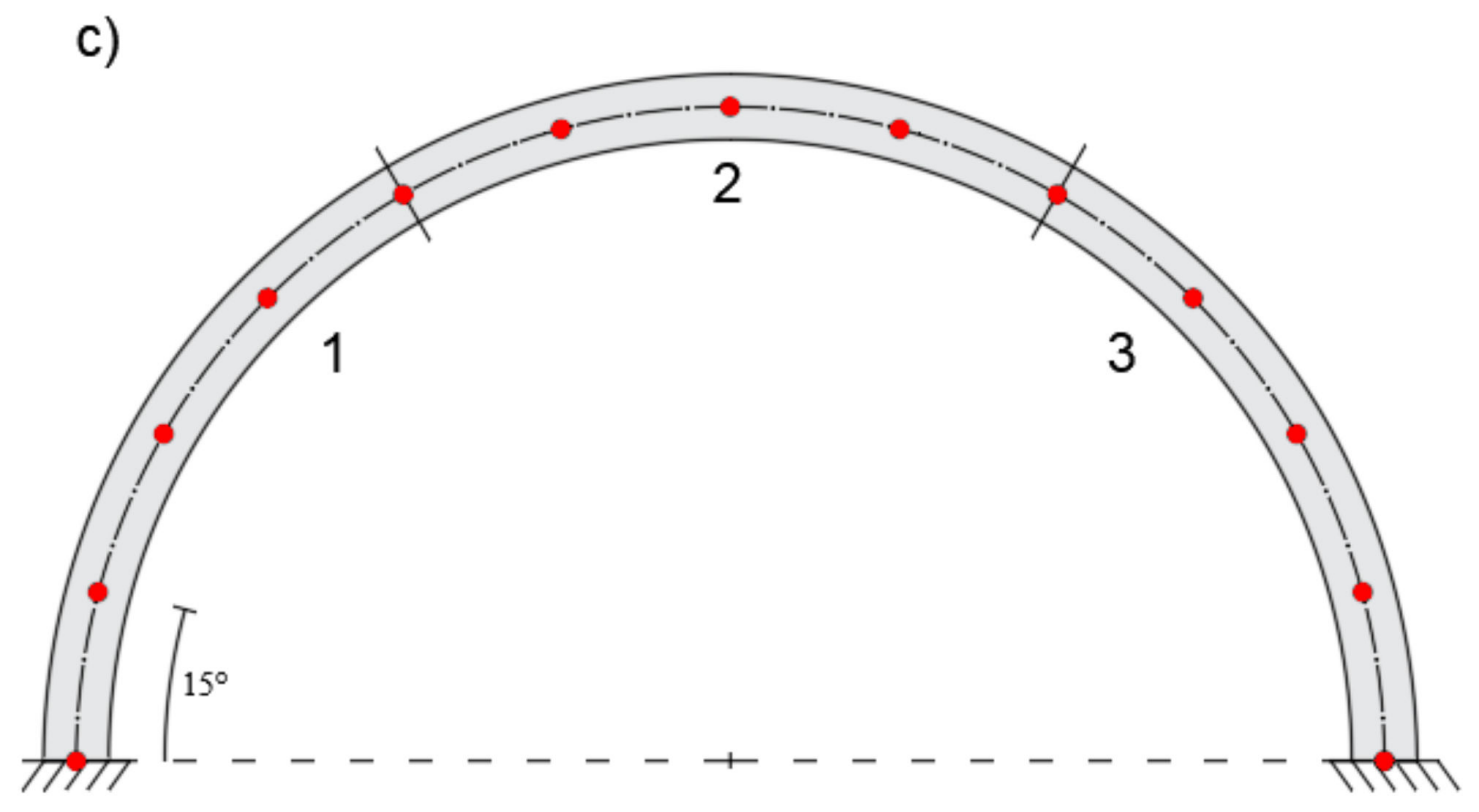
(**a**) Modeling with one, (**b**) two, and (**c**) three inverse elements.

In this case, the iFEM solutions give satisfactory results with only two inverse elements. Figure 10 shows the element discretization and station points for one, two, and three inverse-element meshes.

**Figure 11:**
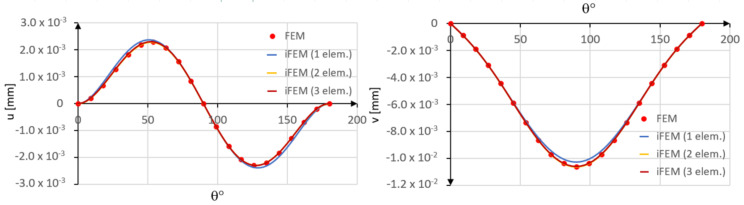
Comparison between iFEM and FEM solutions.

In Figure 11, the iFEM results are compared with those obtained by the direct FEM.

A slight error is observed when a single inverse element is used. In particular, the tangential displacement is slightly overestimated, whereas the radial displacement is slightly underestimated as compared to the direct FEM solution. However, a close agreement is achieved in the case of two inverse elements.

**Figure 12:**
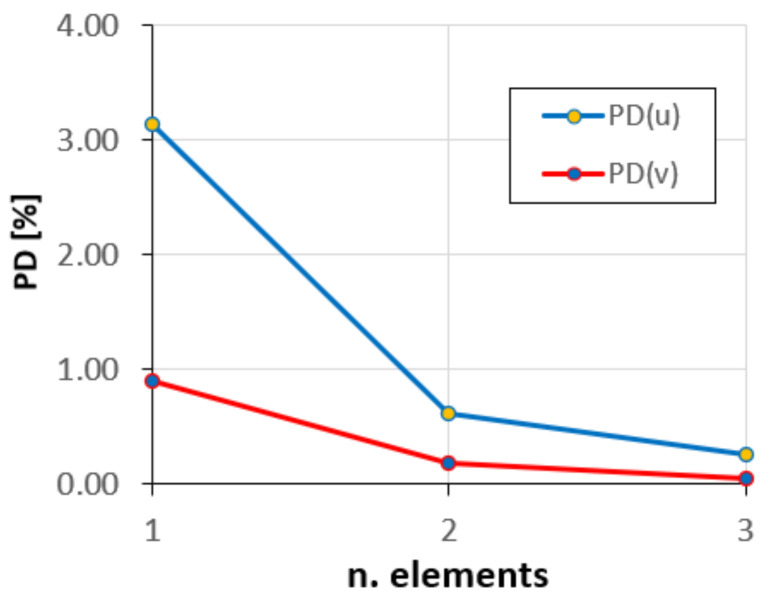
Convergence of the tangential and radial displacements.

The convergence plots of the average percent difference of the tangential PD(u) and radial PD(v) displacements, as the number of elements increases, are shown in Figure 12.

As can be seen, the solutions convergence rapidly, and with only two elements, the error is less than 1%.

### 6.4. A Thin, Deep Arch Clamped at Two Ends

**Figure 13:**
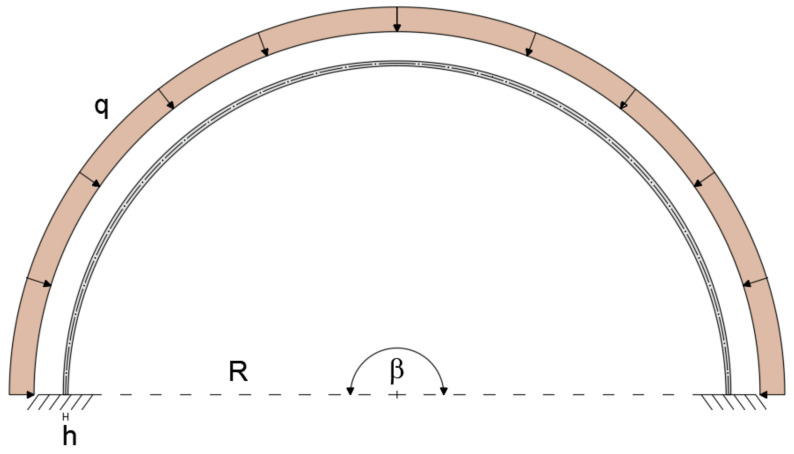
Boundary conditions of the deep, thin arch.

A thin, deep arch with a radius of 5 m, thickness of 0.1 m (R/h = 50), and subtended angle of 180° is subjected to a uniform pressure q. All the other boundary conditions are the same as for the thick arch problem (Figure 13).

**Figure 14:**
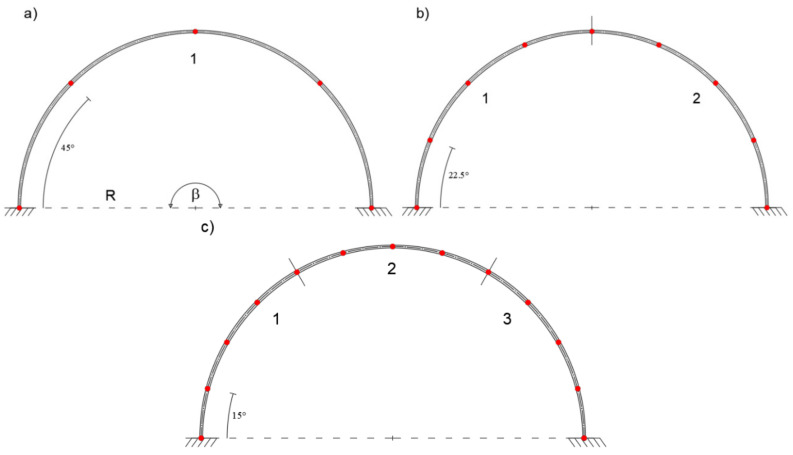
(**a**) Modeling with one, (**b**) two, and (**c**) three inverse elements.

As in the previous example, the arch is discretized using one, two, and three inverse finite elements (Figure 14), which allows assessing the convergence of the reconstructed displacement field.

**Figure 15:**
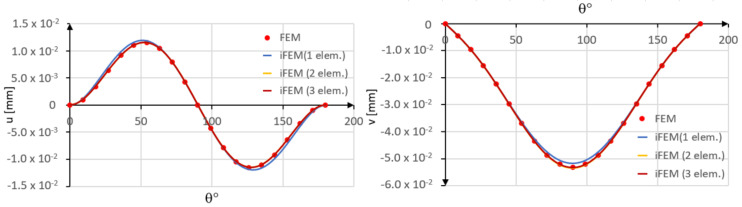
Comparison between iFEM and FEM solutions.

In Figure 15, the tangential u and radial v displacements are plotted versus the angular coordinate.

**Figure 16:**
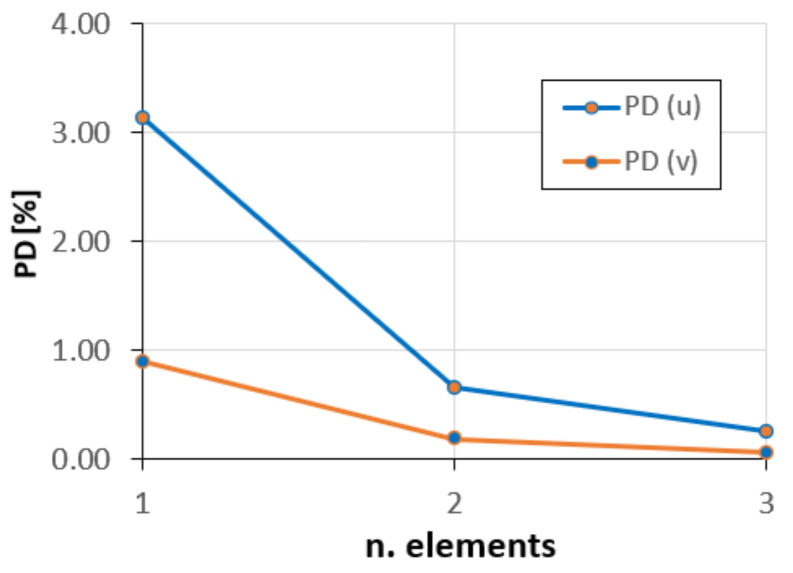
Convergence diagram.

In Figure 16, the PD(u) and PD(v), corresponding to the u and v variables, are plotted against the number of elements in the iFEM discretization.

It is evident from the validation studies that the present inverse element is well suited for modeling both thin and thick curved beams, enabling accurate reconstruction of the displacement field with only a few inverse finite elements.

## 7. Conclusions

The inverse Finite Element Method (iFEM) is a robust and efficient computational method that is designed to perform shape-sensing analysis on a wide range of structures. Based on a variational principle that compares the analytic and measured section strains in a least-square sense, iFEM reconstructs the full-field displacements and strains using only the discrete strain-sensor measurements and structural topology. In this effort, the iFEM framework was used to develop a higher-order curved inverse beam element that was subsequently applied to study the shape sensing of circular arches. Due to the curved geometry, the inverse element was designed with reference to rigid-body modes, bending–membrane coupling, and membrane locking. To this end, the quintic interpolations were chosen in defining the shape functions, resulting in a two-node element with six degrees of freedom at each node. Linear strain–displacement relations of Bernoulli–Euler curved beam theory including membrane and bending deformations were considered without invoking material mechanical properties and load conditions. The shape sensing capability for curved elements was demonstrated on a simple cantilevered curved beam with different values of thickness, radius, and subtended angle. To simulate the in situ strain sensors and provide the reference displacement field, a direct FEM analysis was performed by adopting a high-fidelity mesh. The iFEM shape sensing analysis highlighted the efficiency and effectiveness in predicting the structural response of arch structures. Accurate predictions were obtained by considering few elements and station points without showing locking and convergence problems. The full-field reconstruction of the strain field also implies knowledge of the stress field, thus allowing for real-time damage predictions of curved structures. Future work will examine the effectiveness of the curved inverse beam element for the cases of more complex loading as in geotechnical or aerodynamic configurations. Furthermore, on-field and experimental tests will be performed in order to verify the effectiveness of the present iFEM formulation when dealing with actual noisy data and localized strains.

## Appendix A

The second-order Hermite polynomials, which referred to the one-dimensional case with two interpolation points, can be expressed as follows:

(A1) $$f\left(\xi \right)=\overset{2}{\underset{i=1}{\sum }}\overset{2}{\underset{k=0}{\sum }}H_{ki}^{\left(2\right)}\left(\xi \right)f_{i}^{\left(k\right)}$$

where f(ξ) represents the generic functions to be interpolated, the subscript i refers to the interpolation points where the function f(ξ) is defined, the subscript *k* concerns the derivative degree, and $H_{\mathrm{ki}}^{\left(2\right)}\left(\xi \right)$ represents the quintic Hermite polynomials given by

(A2) $$\frac{d^{r}\;H_{ki}^{\left(2\right)}}{{d\xi }^{r}}\left(\xi_{p}\right)=\delta_{\mathrm{ip}}{\;\delta }_{\mathrm{kr}}.$$

Considering the combinations between p (p = 1, 2) and r (r = 0, 1, 2), Equation (A2) gives the six conditions to calculate the unknown coefficients of the generic polynomial function

(A3) $$H_{\mathrm{ki}}^{\left(2\right)}\left(\xi \right)=a_{1}+a_{2}\xi +a_{3}\xi^{2}+a_{4}\xi^{3}+a_{5}\xi^{4}+a_{6}\xi^{5}.$$

By imposing the conditions given by Equation (A2), the following quintic Hermite polynomials can be obtained

(A4) $$H_{01}^{\left(2\right)}\left(\xi \right)=1-10\xi^{3}+15\xi^{4}-6\xi^{5}$$

(A5) $$H_{11}^{\left(2\right)}\left(\xi \right)=L\left(\xi -6\xi^{3}+8\xi^{4}-3\xi^{5}\right)$$

(A6) $$H_{21}^{\left(2\right)}\left(\xi \right)=\frac{L^{2}}{2}\left(\xi^{2}-3\xi^{3}+3\xi^{4}-\xi^{5}\right)$$

(A7) $$H_{02}^{\left(2\right)}\left(\xi \right)=10\xi^{3}-15\xi^{4}+6\xi^{5}$$

(A8) $$H_{12}^{\left(2\right)}\left(\xi \right)=L\left(-4\xi^{3}+7\xi^{4}-3\xi^{5}\right)$$

(A9) $$H_{22}^{\left(2\right)}\left(\xi \right)=\frac{L^{2}}{2}\left(\xi^{3}-2\xi^{4}+\xi^{5}\right)$$

where ξ = ϕ/β ∈ [0, 1] is the non-dimensional angular coordinate with ϕ ∈ [0, β].
